# Validation of the Japanese Association for the Surgery of Trauma Type 3 Subclassification of Renal Trauma Using the Japan Trauma Data Bank

**DOI:** 10.1111/iju.70632

**Published:** 2026-09-09

**Authors:** Masato Yanagi, Ryuta Nakae, Hayato Takeda, Yuki Endo, Yuka Toyama, Shoji Yokobori, Jun Akatsuka

**Affiliations:** ^1^ Department of Urology Nippon Medical School Hospital Tokyo Japan; ^2^ Department of Emergency and Critical Care Medicine Nippon Medical School Tokyo Japan

**Keywords:** blunt renal trauma, Japanese Association for the Surgery of trauma, operative management, retrospective studies, trauma data bank

## Abstract

**Objectives:**

The Japanese Association for the Surgery of Trauma (JAST) renal trauma classification is widely used in Japan but has not been validated using nationwide multicenter data. This study aimed to assess its clinical validity in blunt renal trauma.

**Methods:**

We conducted a retrospective cohort study using 2019–2024 data from the Japan Trauma Data Bank (JTDB), a nationwide multicenter trauma registry. Among 1256 renal trauma patients in the JTDB, we included those with isolated JAST Type 3 blunt renal trauma (severe parenchymal renal trauma) and classified them as Type 3a or 3b. Multivariable logistic regression was used to identify factors associated with operative management (OM).

**Results:**

Among 219 patients (Type 3a trauma, *n* = 136; Type 3b trauma, *n* = 83), OM was more frequent in Type 3b (14.5% vs. 2.9%, *p* = 0.002). In multivariable analysis, shock on arrival (odds ratio [OR], 7.96; 95% confidence interval [CI], 2.11–30.08; *p* = 0.002) and Type 3b trauma (OR, 4.49; 95% CI, 1.35–14.96; *p* = 0.023) were independently associated with OM.

**Conclusions:**

This nationwide multicenter cohort study demonstrated that JAST Type 3b trauma was independently associated with OM in patients with blunt renal trauma. The JAST Type 3 subclassification provides clinically relevant information for predicting the need for OM.

## 1 Introduction

Renal trauma is among the most common types of genitourinary trauma and accounts for a notable proportion of abdominal organ injury in patients with trauma [1]. Over recent decades, management has shifted toward nonoperative management (NOM), particularly in hemodynamically stable patients, supported by advances in adjunctive interventions such as angioembolization [2, 3, 4, 5, 6]. Consequently, most renal traumas, including selected severe traumas, can now be managed conservatively. Nonetheless, a subset of patients still requires operative management (OM), particularly when there is ongoing hemorrhage, hemodynamic instability, or severe parenchymal trauma [7]. Because delayed surgical intervention may worsen outcomes, early identification of patients at high risk of OM remains an important clinical challenge in renal trauma management.

Several classification systems have been developed to grade renal trauma. Among these, the American Association for the Surgery of Trauma (AAST) classification is the most widely used internationally and has been shown to correlate with clinical outcomes and management strategies [8, 9, 10]. In Japan, however, the Japanese Association for the Surgery of Trauma (JAST) classification has long been used in clinical practice [11, 12]. The JAST classification was developed to reflect the clinical characteristics of renal trauma care in Japan, where blunt trauma predominates and computed tomography (CT)‐based decision‐making has created the need for a detailed, imaging‐based anatomical classification system. The 2008 revision of the JAST classification categorizes renal trauma into types 1–3 according to trauma morphology and the severity of parenchymal damage, with additional subclassifications (e.g., 1a, 1b, 3a, and 3b) to further describe anatomical trauma patterns [11]. Among these, Type 3 traumas are clinically important because they represent deep parenchymal traumas for which intervention is more frequently considered, and subclassification into Types 3a and 3b may allow more precise stratification of patients at risk for OM. However, the clinical validity of this subclassification has not been adequately evaluated using large‐scale nationwide data, and evidence supporting its use in current Japanese urotrauma guidelines is lacking [12].

Therefore, this study aimed to assess the clinical validity of the JAST subclassification of Type 3 renal trauma in predicting OM in patients with blunt renal trauma using data from the Japan Trauma Data Bank (JTDB), a nationwide trauma registry.

## 2 Methods

### 2.1 Study Design and Setting

This nationwide retrospective cohort study was conducted using data from the JTDB. The JTDB is a nationwide hospital‐based trauma registry established in 2003 by the Japanese Association for the Surgery of Trauma [13]. Clinical data are prospectively collected via a web‐based system from participating trauma and emergency centers across Japan. In 2022, 302 major emergency medical institutions participated in the registry [13].

Since January 2019, renal trauma has been classified in the JTDB according to the most recent (2008) revision of the JAST classification (Table 1). Therefore, this study included patients registered between 2019 and 2024 to ensure uniform trauma classification and a consistent procedural data structure. The study protocol was approved by the Institutional Ethics Committee of Nippon Medical School. The requirement for informed consent was waived because all analyses were conducted using anonymized registry data (approval no. S‐2025‐443).

**TABLE 1:** Comparison of JAST classification 2008 and AAST classification 2018.

Type	Description	Alternative AAST grade
1a	Subcapsular trauma	1
1b	Intraparenchymal hematoma	1
2	Superficial trauma	2 or 3
3a	Simple deep trauma: More than half the depth of parenchymal laceration	3 or 4
3b	Complex deep trauma: Transected or shattered kidney	4 or 5
Appendix		
・PV: Pedicle vessel trauma		5
・Degrees of expanding hematoma		
H1: Hematoma within Gerota's fascia		Not applicable
H2: Hematoma beyond Gerota's fascia		Not applicable
・Presence of urinary extravasation beyond Gerota's fascia		
U1:Urinary extravasation within Gerota's fascia		Not applicable
U2:Urinary extravasation beyond Gerota's fascia		Not applicable
・Penetrating trauma		
S: Stab wound		Not applicable
G: Gunshot wound		Not applicable

*Note:* AAST, American Association for the Surgery of Trauma.

### 2.2 Study Population

Patients with isolated blunt renal trauma registered in the JTDB between 2019 and 2024 were analyzed. Since January 2019, detailed organ‐specific procedural data have not been available; instead, procedures have been categorized into broader groups, such as abdominal surgery, angiography, and transcatheter arterial embolization. Because kidney‐specific interventions cannot be reliably identified in patients with multiple organ trauma, the study population was restricted to patients with isolated renal trauma to minimize outcome misclassification and ensure that the recorded interventions were attributable to renal trauma. Isolated renal trauma was defined as renal injury without concomitant injuries to other organs recorded in the JAST organ injury classification.

**FIGURE 1:**
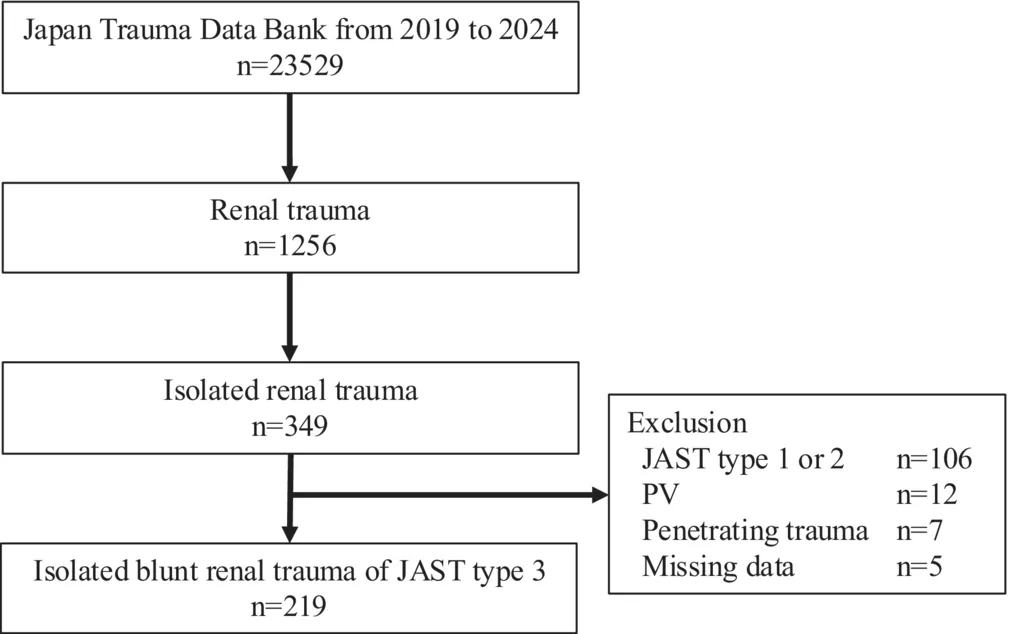
Patient flowchart. JAST, Japanese Association for the Surgery of Trauma.

The patient flowchart is shown in Figure 1. Patients with JAST Type 1 or 2 trauma were excluded because these injuries are generally managed non‐operatively [3, 4]. Patients with pedicle vessel trauma (PV) were also excluded because they are typically considered absolute indications for surgical intervention [5]. Patients with penetrating trauma were excluded. Additionally, a small number of patients (*n* = 5) with missing data on key baseline variables, including age, sex, or vital signs on hospital arrival, were excluded. We focused on patients with JAST Type 3 blunt renal trauma, representing clinically significant renal trauma for which operative management may be required.

### 2.3 Definitions and Variables

**FIGURE 2:**
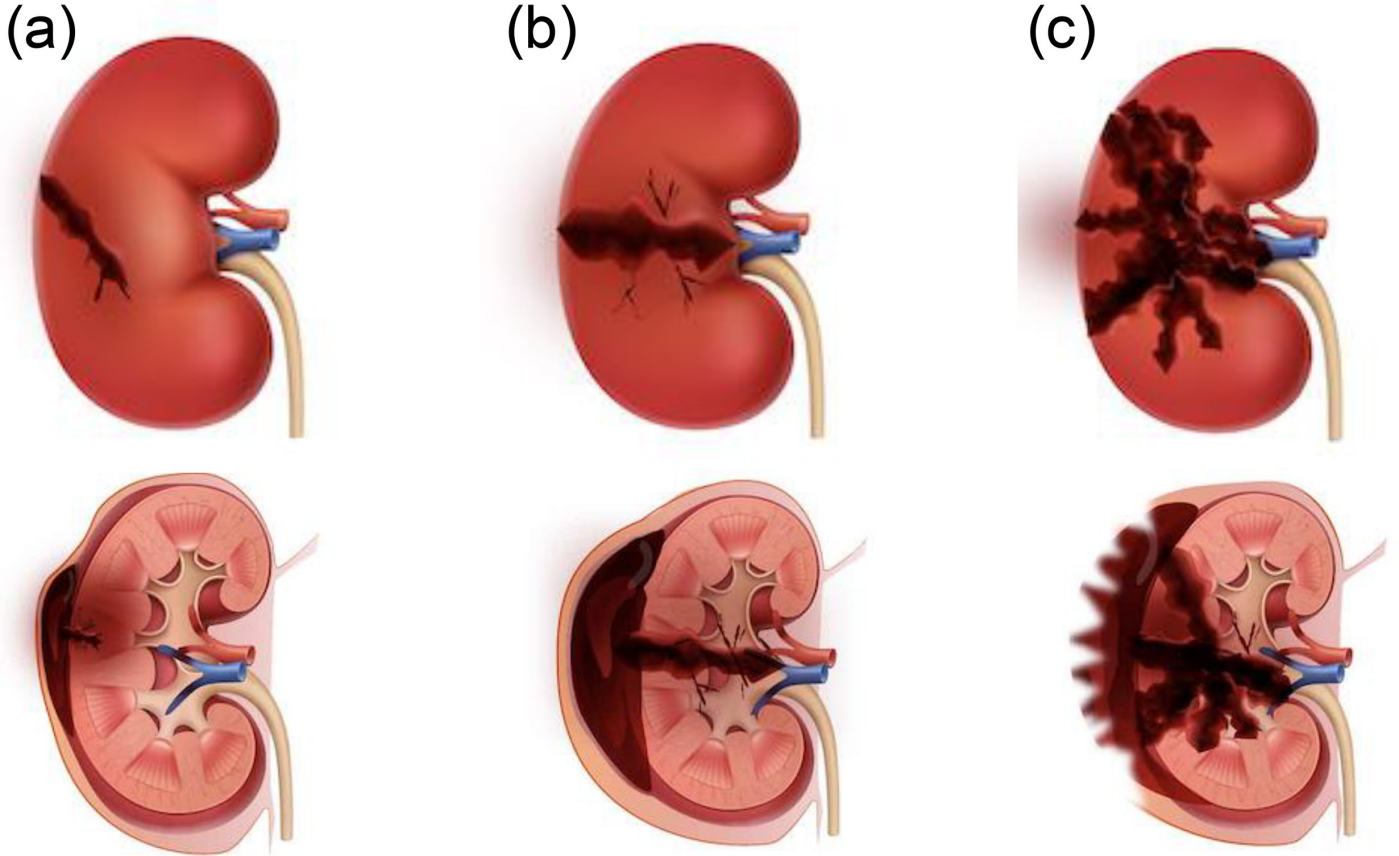
Renal trauma morphology, JAST Type 3. (a) JAST Type 3a: Simple deep trauma; (b) JAST Type 3b: Transected kidney; (c) JAST Type 3b: Shattered kidney.

Renal trauma was classified according to the JAST 2008 classification (Table 1, Figure 2), which is primarily based on contrast‐enhanced computed tomography findings [11]. Classification was prospectively recorded in the registry by the attending physicians at each participating institution. JAST Type 3 was defined as a deep parenchymal laceration involving > 50% of the renal parenchymal depth. Type 3 is further subdivided into Types 3a and 3b: Type 3a represents simple deep lacerations, whereas Type 3b includes complex trauma, such as transected or shattered kidneys. The JAST classification system also includes additional modifiers. The presence of pedicle vessel trauma was denoted as “PV.” The extent of the perirenal hematoma was categorized using the H factor (H1: confined within Gerota's fascia; H2: extending beyond Gerota's fascia), and urinary extravasation was categorized using the U factor (U1: within Gerota's fascia; U2: beyond Gerota's fascia).

Data for eligible patients were extracted from the JTDB database based on trauma classification, mechanism of trauma, and recorded procedural information. Extracted variables included age, sex, mechanism of trauma, use of antiplatelet or anticoagulant medications, vital signs at hospital arrival, JAST classification (including subtype and modifiers), transfusion within 24 h after arrival, angiography, transcatheter arterial embolization (TAE), OM, in‐hospital mortality, and hospital length of stay. Since detailed organ‐specific operative procedures are not available in the JTDB after 2019, OM was defined as any recorded abdominal surgical intervention in patients with isolated renal trauma. Shock was defined as systolic blood pressure < 90 mmHg at hospital arrival, consistent with trauma guidelines.

### 2.4 Primary Outcomes

The primary outcome was OM, defined as any recorded abdominal surgical intervention in the JTDB.

### 2.5 Statistical Analysis

Continuous variables were compared using the Mann–Whitney U test, and categorical variables were compared using the chi‐square test. Univariable logistic regression analyses were first performed to identify factors associated with OM. Because the temporal sequence between transfusion within 24 h and OM could not be determined from the JTDB, transfusion within 24 h was not included in the multivariable model. Variables with a *p*‐value < 0.05 in the univariable analysis were subsequently included in the multivariable logistic regression model to identify independent predictors of OM. Odds ratios (ORs) with 95% confidence intervals (CIs) were calculated. As an exploratory analysis, the association between the U factor and OM was evaluated among patients with available U‐factor data using Fisher's exact test. A *p*‐value < 0.05 was considered statistically significant.

## 3 Results

### 3.1 Patient Characteristics

Overall, 219 patients with JAST Type 3 isolated blunt renal trauma were included in the analysis: 136 with Type 3a trauma and 83 with Type 3b trauma. Patient characteristics are summarized in Table 2. No significant between‐group differences were observed in age (median, 34 years [interquartile range 18–64] vs. 40 years [18–63], *p* = 0.356), sex distribution (male, 72.1% vs. 73.5%, *p* = 0.892), or use of antiplatelet or anticoagulant medications (3.7% vs. 4.8%, *p* = 0.682). Trauma mechanisms were broadly similar between groups. Overall, falls were the most common cause (45.7%), followed by traffic accidents (29.2%) and other mechanisms (25.1%), and their distribution did not differ markedly between JAST Types 3a and 3b. Shock on hospital arrival was significantly more frequent in the Type 3b group than in the Type 3a group (10.8% vs. 2.9%, *p* = 0.018). Type 3b was associated with a higher proportion of H2 hematoma extension than Type 3a (41.0% vs. 17.6%, *p* < 0.001).

**TABLE 2:** Patient characteristics.

*n*	Total	JAST Type 3a	JAST Type 3b	*p*
No. of patients	219	136	83	
Age (years): median (IQR)	38 (18–63)	34 (18–64)	40 (18–63)	0.356
Gender (%)				0.892
Male	159 (72.6)	98 (72.1)	61 (73.5)	
Female	60 (27.4)	38 (27.9)	22 (26.5)	
Oral administration of antiplatelet or anticoagulant drugs	9 (4.1)	5 (3.7)	4 (4.8)	0.682
Mechanism (%)				NA
Traffic accident	64 (29.2)	46 (33.8)	18 (21.7)	
Fall	100 (45.7)	59 (43.4)	41 (49.4)	
Others	55 (25.1)	31 (22.8)	24 (28.9)	
Shock at arrival (%)	13 (5.9)	4 (2.9)	9 (10.8)	*0.018
H factor (%)				* < 0.001
H1	75 (34.2)	58 (42.6)	17 (20.5)	
H2	58 (26.5)	24 (17.6)	34 (41.0)	
Unknown	86 (39.3)	54 (39.7)	32 (38.6)	
U factor (%)				NA
U1	31 (14.2)	18 (13.2)	13 (15.7)	
U2	9 (4.1)	2 (1.5)	7 (8.4)	
Unknown	179 (81.7)	116 (85.3)	63 (75.9)	
Transfusion requirement within 24 h from arrival (%)	44 (20.1)	21 (15.4)	23 (27.7)	*0.030
Angiography (%)	79 (36.1)	39 (28.7)	40 (48.2)	*0.004
TAE (%)	57 (26.0)	26 (19.1)	31 (37.3)	*0.003
OM (%)	16 (7.3)	4 (2.9)	12 (14.5)	*0.002
Dead	2 (0.9)	1 (0.7)	1 (1.2)	0.727
Hospital stays (days): median (IQR)	11 (7–17)	10 (6–15)	14 (10–20)	* < 0.001

Abbreviations: IQR, interquartile range; JAST, Japanese Association for the Surgery of Trauma; NA, not available; OM, operative management; TAE, transcatheter arterial embolization.

### 3.2 Management and Interventions

Patients with JAST Type 3b required more intensive interventions than those with JAST Type 3a. The 24 h transfusion rate was significantly higher in the Type 3b group (27.7% vs. 15.4%, *p* = 0.030). Similarly, angiography (48.2% vs. 28.7%, *p* = 0.004) and transcatheter arterial embolization (37.3% vs. 19.1%, *p* = 0.003) were performed more frequently in patients with JAST Type 3b. OM was performed in 16 patients (7.3%). The OM rate was significantly higher in patients with JAST Type 3b than in those with JAST Type 3a (14.5% vs. 2.9%, *p* = 0.002). Among the four patients with JAST Type 3a who underwent OM, none presented with shock on arrival, whereas three patients received blood transfusions within 24 h of hospital arrival. Further details regarding the indications for OM could not be determined from the JTDB. In an exploratory analysis restricted to the 40 patients with available U‐factor data, OM was performed in 4 of 31 patients (12.9%) with U1 and in none of the 9 patients (0%) with U2; there was no significant difference between the groups (Fisher's exact test, *p* = 0.557; Table S1). Additionally, there were two (0.9%) in‐hospital deaths, one in each group (JAST Types 3a and 3b). Hospital stay was significantly longer in patients with JAST Type 3b than in those with JAST Type 3a (*p* < 0.001).

### 3.3 Predictors of OM

In the univariable analysis, shock at arrival (OR 11.08, 95% CI 3.11–39.52, *p* < 0.001), JAST Type 3b (OR 5.58, 95% CI 1.76–17.93, *p* = 0.004), and transfusion within 24 h (OR 11.33, 95% CI 3.69–34.76, *p* < 0.001) were significantly associated with OM (Table 3). In the multivariable logistic regression analysis, shock at arrival (OR 7.96, 95% CI 2.11–30.08, *p* = 0.002) and JAST Type 3b trauma (OR 4.49, 95% CI 1.35–14.96, *p* = 0.023) remained independently associated with OM (Table 3).

**TABLE 3:** Risk factors for OM as determined by univariate and multivariate analyses.

	Univariable analysis			Multivariable analysis		
	OR	95% CI	*p*	OR	95% CI	*p*
≥ 75 years old	0.79	0.17–3.66	0.628			
Man	1.14	0.356–3.69	0.823			
Oral administration of antiplatelet or anticoagulant drugs	1.63	0.19–13.87	0.657			
Shock at arrival	11.08	3.11–39.52	* < 0.001	7.964	2.11–30.08	*0.002
JAST Type 3b	5.58	1.76–17.93	*0.004	4.49	1.35–14.96	*0.023
H2	2.32	0.82–6.54	0.112			
Transfusion requirement within 24 h of arrival	11.33	3.69–34.76	* < 0.001			

Abbreviations: CI, confidence interval; JAST, Japanese Association for the Surgery of Trauma; OM, operative management; OR, odds ratio.

## 4 Discussion

Using a nationwide trauma database in Japan, we examined the association between the JAST subclassification and OM among patients with isolated blunt renal trauma classified as JAST Type 3. We found that JAST Type 3b was an independent predictor of OM (OR 4.49, 95% CI 1.35–14.96, *p* = 0.023). To our knowledge, this is the first nationwide study evaluating the association between JAST subclassifications and OM. Our findings support the clinical utility of the JAST classification in guiding early treatment decisions for renal trauma.

The incidence of renal trauma in Japan is relatively low, at approximately 2.06 cases per 100 000 person‐years, with males accounting for 72%–74.2% of cases and a median age of 41–43 years [12, 14]. Because of strict firearm regulations, approximately 95% of renal injuries in Japan are caused by blunt trauma, with traffic accidents being the most common mechanism of trauma [12, 14]. Although most blunt renal injuries can now be managed successfully with NOM, a subset of patients with JAST Type 3 injuries still require OM, making early identification of high‐risk patients an important clinical challenge [12]. Nonetheless, the declining incidence of severe traffic‐related trauma has made it increasingly difficult for individual institutions to accumulate a sufficient number of severe renal trauma cases, such as JAST Type 3 injuries, for meaningful analysis. Consequently, most evidence incorporated into the current Japanese urotrauma guideline is based on the AAST classification, whereas the clinical validity of the 2008 JAST classification has not been adequately evaluated [12]. Further validation of the JAST classification is, therefore, warranted to establish evidence supporting the classification system routinely used in Japan. Fortunately, the JTDB adopted the 2008 JAST classification in 2019, enabling nationwide analyses based on the JAST subclassification. Accordingly, we conducted a nationwide study using the JTDB to examine the clinical significance of the long‐debated JAST Type 3a/3b subclassification. By analyzing JTDB data from 2019 to 2024, we identified 219 patients with isolated blunt renal trauma classified as JAST Type 3, providing a sufficiently large cohort for robust analysis.

Our findings are consistent with those of previous studies using the AAST classification, which showed that greater trauma severity was associated with the need for OM. In a prospective cohort study of 151 patients with high‐grade blunt renal trauma treated at a Level I trauma center outside Japan, Lanchon et al. reported that AAST Grade V (OR 7.36, *p* = 0.01) and hemodynamic instability (OR 4.18, *p* = 0.04) were independent predictors of OM [15]. Thus, patients with severe renal trauma may be more likely to require OM. Similarly, Nakao et al. analyzed 3 550 renal trauma cases from the JTDB and demonstrated that AAST grades IV and V were strong predictors of nephrectomy [3]. Their findings indicated that increased trauma severity was associated with a greater likelihood of renal preservation failure. Using JTDB data classified according to the JAST system, we performed a multivariable analysis with OM as the primary outcome. Our results demonstrate that the association between trauma severity and the need for OM, previously established using the AAST classification, is also evident in the widely used JAST classification in Japan. Although the unique structural features of the JAST classification, including its separate H and U modifiers, have been recognized internationally, their large‐scale clinical validation has been lacking [8]. Our findings help fill this important evidence gap by providing the first nationwide validation of the JAST Type 3 subclassification.

Among the 219 patients with isolated blunt renal trauma classified as JAST Type 3, 83 (37.9%) were classified as JAST Type 3b, indicating that Type 3b is common and represents an important trauma pattern in routine trauma practice. JAST Type 3b is radiographically defined as a shattered kidney or renal transection and is characterized by more extensive parenchymal destruction and vascular injury than Type 3a. Consistent with this definition, patients with JAST Type 3b in our cohort had significantly higher rates of shock on arrival, blood transfusion within 24 h, angiography, and TAE, as well as a higher frequency of OM. Therefore, JAST Type 3b may not only reflect a radiologic classification but also a more severe clinical condition associated with physiological deterioration and the need for invasive interventions. Nevertheless, only 12 patients (14.5%) with JAST Type 3b ultimately required OM, whereas approximately 85% of patients were successfully managed with NOM. This observation suggests that even patients with JAST Type 3b can often be managed nonoperatively when appropriate patient selection and contemporary trauma management strategies are applied.

Here, shock on arrival was the strongest predictor of OM (OR 7.96, 95% CI 2.11–30.08, *p* = 0.002), reflecting the importance of hemodynamic status in treatment decision‐making for renal trauma. In contrast, the JAST classification is an imaging‐based system that can be uniformly assessed during the initial evaluation using contrast‐enhanced CT findings. Notably, JAST Type 3b remained independently associated with OM even after adjustment for shock on arrival. Therefore, the JAST classification may provide clinically relevant anatomical information beyond hemodynamic status and serve as a useful imaging biomarker for estimating the risk of OM when combined with other clinical information available during the initial assessment.

This study has some limitations. First, this was a retrospective study, and residual confounding from unmeasured variables could not be completely ruled out. Second, since 2019, the JTDB has not recorded detailed information on specific procedures, and interventions have been broadly categorized by body region. Therefore, it is difficult to determine whether a given intervention was performed specifically for renal trauma in patients with multiple injuries. To minimize potential misclassification bias, we restricted our analysis to patients with isolated renal trauma. Third, detailed clinical information, including transfusion indications and exact timing, specific contrast‐enhanced CT findings, and operative details, was not available in the JTDB. Therefore, the temporal relationship between blood transfusion and operative management could not be determined, and causality could not be inferred. In addition, detailed organ‐specific operative procedures were unavailable, and operative management was identified based on broader procedural categories. Fourth, U factor was unavailable for the majority of the patients, and only a limited number had complete U‐factor data. Therefore, this exploratory analysis of the association between the U factor and OM was limited by the substantial proportion of missing data and small number of complete cases. Further, specific urinary collecting system injuries (e.g., ureteropelvic junction or renal pelvic rupture) could not be identified from the JTDB, even though these injuries may require OM in some patients. Future studies using datasets with more complete imaging information are warranted to further clarify the significance of urinary collecting system injuries and the U factor in terms of predicting OM. Despite these limitations, this study provides the first nationwide clinical validation of the JAST Type 3 subclassification by demonstrating an independent association between JAST Type 3b trauma and OM. Notably, only 14.5% of our patients with Type 3b trauma ultimately required surgery, whereas 85.5% were successfully managed with NOM. These findings indicate that the current JAST Type 3 subclassification is useful for identifying patients at increased risk of OM, while highlighting that most Type 3b injuries can be managed conservatively using appropriate patient selection and contemporary trauma management. The relatively low OM rate among our patients with Type 3b trauma also suggests that further refinement of risk stratification may be warranted. In future revisions of the JAST classification, subdivision of the current Type 3b category into transected and shattered kidneys should be considered. Similarly, the incorporation of active contrast extravasation on CT as an additional imaging modifier may also be considered in future revisions, in line with the recent evidence‐based update of the AAST classification [16].

In conclusion, this nationwide multicenter cohort study demonstrated that JAST Type 3b trauma was independently associated with OM in patients with blunt renal trauma. The JAST Type 3 subclassification provides clinically relevant information for predicting the need for OM and may assist in early management decisions.

## Author Contributions

**Yuka Toyama:** data curation, investigation. **Ryuta Nakae:** data curation, investigation, writing – review and editing. **Hayato Takeda:** data curation, investigation. **Yuki Endo:** data curation, investigation. **Masato Yanagi:** conceptualization, methodology, data curation, investigation, validation, writing – original draft, writing – review and editing. **Shoji Yokobori:** investigation, writing – review and editing, supervision. **Jun Akatsuka:** investigation, writing – review and editing, resources, supervision.

## Ethics Statement

This study was performed in line with the principles of the Declaration of Helsinki. The Ethics Committee at Nippon Medical School Hospital approved this study (approval no. S‐2025‐443).

## Consent

The requirement for informed consent was waived due to the retrospective nature of the study using a nationwide database.

## Conflicts of Interest

The authors declare no conflicts of interest.

## Supporting information


**Table S1:** Exploratory analysis of the association between U factor and operative management.

## Data Availability

Research data are not shared.
