# Supplementary material for: Validation of the Japanese Association for the Surgery of Trauma Type 3 Subclassification of Renal Trauma Using the Japan Trauma Data Bank
Source: Int J Urol. 2026 Sep 9;33(9):e70632. doi: 10.1111/iju.70632 (PMC13558463; doi:10.1111/iju.70632)
Supplement: Supplementary file 1 [file IJU-33-0-s001.docx]

**Supplementary Table S1. Exploratory analysis of the association between U factor and operative management**

|  | NOM, n (%) | OM, n (%) | P value |
| --- | --- | --- | --- |
| U factor |  |  | 0.557 |
| U1 | 27 (87.1) | 4 (12.9) |  |
| U2 | 9 (100) | 0 (0) |  |

NOM, Nonoperative management; OM, Operative management
